# (*E*)-2-Cyano-3-[4-(dimethyl­amino)phen­yl]-*N*-phenyl­prop-2-enamide

**DOI:** 10.1107/S1600536809017681

**Published:** 2009-05-14

**Authors:** Abdullah Mohamed Asiri, Mehmet Akkurt, Salman A. Khan, Islam Ullah Khan, Muhammad Nadeem Arshad

**Affiliations:** a Chemistry Department, Faculty of Science, King Abdul-Aziz University, PO Box 80203, Jeddah 21589, Saudi Arabia; b Department of Physics, Faculty of Arts and Sciences, Erciyes University, 38039 Kayseri, Turkey; c Department of Chemistry, Government College University, Lahore, Pakistan

## Abstract

In the title compound, C_18_H_17_N_3_O, the dihedral angle between the phenyl and benzene rings is 11.22 (14)°. Apart from the methyl H atoms, the mol­ecule is close to planar, with a maximum deviation of 0.145 (3) Å. Intra­molecular C—H⋯O and C—H⋯N inter­actions occur. In the crystal, inversion dimers linked by pairs of N—H⋯N hydrogen bonds occur, resulting in an *R*_2_^2^(12) ring motif. Further C—H⋯N and C—H⋯O bonds generate *R*_1_^2^(7) and *R*_2_^2^(22) motifs and a C—H⋯π inter­action also occurs.

## Related literature

For background on the properties and uses of organic dyes, see: Grabowski *et al.* (2003 ▶); Guo *et al.* (2007 ▶); Kwak *et al.* (2008 ▶); Moylan *et al.* (1996 ▶). For reference structural data, see Allen *et al.* (1987 ▶). For graph-set terminology, see: Bernstein *et al.* (1995 ▶).
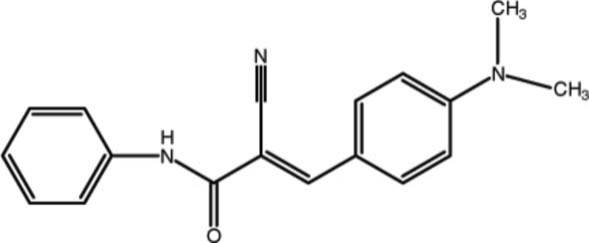


## Experimental

### 

#### Crystal data

C_18_H_17_N_3_O
*M* *_r_* = 291.35
Monoclinic, 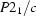

*a* = 12.0639 (19) Å
*b* = 19.983 (3) Å
*c* = 6.3960 (9) Å
β = 94.870 (6)°
*V* = 1536.3 (4) Å^3^
*Z* = 4
Mo *K*α radiation
μ = 0.08 mm^−1^
*T* = 296 K
0.44 × 0.09 × 0.07 mm

#### Data collection

Bruker Kappa APEXII CCD diffractometer
Absorption correction: none
15701 measured reflections
3484 independent reflections
1380 reflections with *I* > 2σ(*I*)
*R*_int_ = 0.082

#### Refinement

*R*[*F* ^2^ > 2σ(*F* ^2^)] = 0.060
*wR*(*F* ^2^) = 0.165
*S* = 0.97
3484 reflections
202 parameters
H-atom parameters constrained
Δρ_max_ = 0.19 e Å^−3^
Δρ_min_ = −0.16 e Å^−3^

### 

Data collection: *APEX2* (Bruker, 2007 ▶); cell refinement: *SAINT* (Bruker, 2007 ▶); data reduction: *SAINT*; program(s) used to solve structure: *SIR97* (Altomare *et al.*, 1999 ▶); program(s) used to refine structure: *SHELXL97* (Sheldrick, 2008 ▶); molecular graphics: *ORTEP-3 for Windows* (Farrugia, 1997 ▶); software used to prepare material for publication: *WinGX* (Farrugia, 1999 ▶) and *PLATON* (Spek, 2009 ▶).

## Supplementary Material

Crystal structure: contains datablocks global, I. DOI: 10.1107/S1600536809017681/hb2974sup1.cif

Structure factors: contains datablocks I. DOI: 10.1107/S1600536809017681/hb2974Isup2.hkl

Additional supplementary materials:  crystallographic information; 3D view; checkCIF report

## Figures and Tables

**Table 1:** Hydrogen-bond geometry (Å, °)

*D*—H⋯*A*	*D*—H	H⋯*A*	*D*⋯*A*	*D*—H⋯*A*
C5—H5⋯O1	0.93	2.30	2.892 (4)	121
C12—H12⋯N2	0.93	2.61	3.445 (4)	149
N1—H1*A*⋯N2^i^	0.86	2.50	3.245 (3)	146
C1—H1⋯N2^i^	0.93	2.58	3.338 (4)	139
C18—H18*A*⋯O1^ii^	0.96	2.49	3.439 (4)	169
C3—H3⋯*Cg*1^iii^	0.93	2.66	3.514 (3)	152

Symmetry codes: (i) 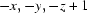; (ii) 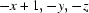; (iii) 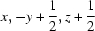. *Cg*1 is the centroid of the C1–C6 phenyl ring.

**Fig. 1.**
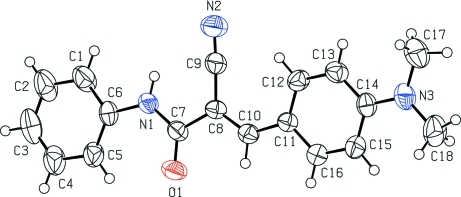
The molecular structure of (I) showing displacement ellipsoids at the 50% probability level. H atoms are drawn as spheres of arbitrary radius.

**Fig. 2.**
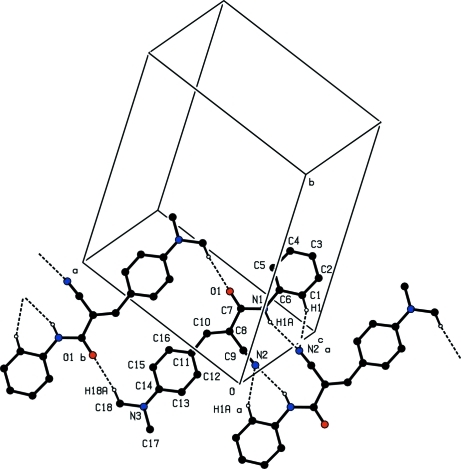
View of the hydrogen bonding interactions (dashed lines) for (I). H atoms not involved in hydrogen bonding have been omitted for clarity. [Symmetry codes: (a) 1–x, –y, 1–z; (b) 1–x, –y, –z].

**Fig. 3.**
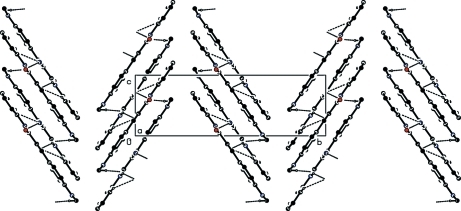
The molecular packing for (I) viewed down the a axis showing the hydrogen bonding interactions (dashed lines). Molecules form a zigzag pattern along the b axis. H atoms not involved in hydrogen bonding have been omitted for clarity.

## supplementary crystallographic information

### Comment

Organic dyes with donor–π-conjugation–acceptor (D–π–A) molecular structure have attracted much attention because of their inherent nonlinear optical characteristics, which are highly sensitive to changes in the external environment such as polarity and pH of media, due to their intrinsic character (*e.g.* Grabowski *et al.*, 2003). They have been intensively developed for applications using as photo-(PL) and electroluminescent (EL) materials in the fields of dye laser (Moylan *et al.*, 1996), fluorescent sonser and logic memory (Guo *et al.*, 2007), and organic light-emitting device (OLED) (Kwak *et al.*, 2008). The title compound, (I) (Fig. 1), is a representative of Push-Pull systems with dimethylamino group as a donor at one end of the conjugated system and cyano and carboonyl as acceptor at the other end.

The molecule of (I) contains a phenyl ring and a benzene ring which makes a dihedral angle of 11.22 (14)°. Except the methyl H atoms, the title molecule is almost planar, with a maximum deviation of 0.145 (3) Å for C12 and C13. The bond lengths and angles are in normal range (Allen *et al.*, 1987). The molecules of the title compound form in which two N—H···N hydrogen bonds. The N—H···N, C—H···N and C—H···O interactions generates *R*_2_^2^(12), *R*_1_^2^(7) and *R*_2_^2^(22) motifs (Fig. 2) (Bernstein *et al.* 1995). Fig. 3 shows the molecular packing for (I) viewed down the *a* axis showing the hydrogen bonding interactions (dashed lines). Molecules form a *zigzag* pattern along the *b* axis.

The crystal structure is stabilized by intermolecular N—H···N, C—H···O and C—H···N hydrogen bonding, and C—H···π interactions (Table 1).

### Experimental

*N*-Phenyl-2-cyanoacetamide (1.60 g, 0.010 mol) and 4-*N*,*N*-dimethylaminobenzaldehyde (1.49 g, 0.010 mol) were dissolved in 50 ml of ethanol then heated to boiling before pipyridine (0.5 ml) was added. The reaction mixture was refluxed for 7 h, cooled then the precipitate was filtered and recrystalized from ethanol to yield red prisms of (I) [yield: 90%, m.p.: 382–384 K]. IR;ν (cm ^-1^): 3348, 2941, 2890 (–C—H), 2198 (CN), 1671 (C=O), 1601 (C=C), 1580 (C=C).

### Refinement

The H atoms were positioned geometrically and treated as riding, with N—H = 0.86 Å and C—H = 0.93–0.96 Å, and refined as riding with *U*_iso_(H) = 1.2 or 1.5*U*_eq_(parent atom).

### Crystal data

**Table:**

C_18_H_17_N_3_O	*F*(000) = 616
*M**_r_* = 291.35	*D*_x_ = 1.260 Mg m^−^^3^
Monoclinic, *P*2_1_/*c*	Mo *K*α radiation, λ = 0.71073 Å
Hall symbol: -P 2ybc	Cell parameters from 1223 reflections
*a* = 12.0639 (19) Å	θ = 3.4–19.8°
*b* = 19.983 (3) Å	µ = 0.08 mm^−^^1^
*c* = 6.3960 (9) Å	*T* = 296 K
β = 94.870 (6)°	Prism, red
*V* = 1536.3 (4) Å^3^	0.44 × 0.09 × 0.07 mm
*Z* = 4	

### Data collection

**Table:**

Bruker Kappa APEXII CCD diffractometer	1380 reflections with *I* > 2σ(*I*)
Radiation source: sealed tube	*R*_int_ = 0.082
graphite	θ_max_ = 27.5°, θ_min_ = 2.0°
φ and ω scans	*h* = −15→15
15701 measured reflections	*k* = −25→25
3484 independent reflections	*l* = −8→5

### Refinement

**Table:**

Refinement on *F*^2^	Secondary atom site location: difference Fourier map
Least-squares matrix: full	Hydrogen site location: inferred from neighbouring sites
*R*[*F*^2^ > 2σ(*F*^2^)] = 0.060	H-atom parameters constrained
*wR*(*F*^2^) = 0.165	*w* = 1/[σ^2^(*F*_o_^2^) + (0.0643*P*)^2^] where *P* = (*F*_o_^2^ + 2*F*_c_^2^)/3
*S* = 0.97	(Δ/σ)_max_ < 0.001
3484 reflections	Δρ_max_ = 0.19 e Å^−^^3^
202 parameters	Δρ_min_ = −0.16 e Å^−^^3^
0 restraints	Extinction correction: *SHELXL97* (Sheldrick, 2008), Fc^*^=KFc[1+0.001XFc^2^Λ^3^/sin(2Θ)]^-1/4^
Primary atom site location: structure-invariant direct methods	Extinction coefficient: 0.0038 (12)

### Special details

**Table:**

Geometry. Bond distances, angles *etc*. have been calculated using the rounded fractional coordinates. All su's are estimated from the variances of the (full) variance-covariance matrix. The cell e.s.d.'s are taken into account in the estimation of distances, angles and torsion angles
Refinement. Refinement on *F*^2^ for ALL reflections except those flagged by the user for potential systematic errors. Weighted *R*-factors *wR* and all goodnesses of fit *S* are based on *F*^2^, conventional *R*-factors *R* are based on *F*, with *F* set to zero for negative *F*^2^. The observed criterion of *F*^2^ > σ(*F*^2^) is used only for calculating -*R*-factor-obs *etc*. and is not relevant to the choice of reflections for refinement. *R*-factors based on *F*^2^ are statistically about twice as large as those based on *F*, and *R*-factors based on ALL data will be even larger.

### Fractional atomic coordinates and isotropic or equivalent isotropic displacement parameters (Å^2^)

**Table:**

	*x*	*y*	*z*	*U*_iso_*/*U*_eq_	
O1	0.37797 (16)	0.07494 (10)	0.5929 (3)	0.0639 (8)	
N1	0.19658 (18)	0.07922 (10)	0.6643 (3)	0.0444 (8)	
N2	0.0364 (2)	−0.00214 (14)	0.2850 (4)	0.0714 (10)	
N3	0.3109 (2)	−0.16820 (12)	−0.4827 (4)	0.0619 (10)	
C1	0.1026 (3)	0.13303 (16)	0.9294 (5)	0.0740 (12)	
C2	0.0994 (3)	0.17166 (17)	1.1086 (6)	0.0876 (17)	
C3	0.1948 (4)	0.19793 (16)	1.2024 (5)	0.0757 (16)	
C4	0.2923 (3)	0.18648 (16)	1.1198 (5)	0.0754 (14)	
C5	0.2976 (3)	0.14743 (15)	0.9411 (5)	0.0615 (11)	
C6	0.2018 (2)	0.12058 (13)	0.8457 (4)	0.0439 (10)	
C7	0.2811 (2)	0.05968 (13)	0.5505 (4)	0.0425 (10)	
C8	0.2467 (2)	0.01696 (13)	0.3648 (4)	0.0391 (9)	
C9	0.1308 (3)	0.00579 (14)	0.3162 (4)	0.0480 (10)	
C10	0.3244 (2)	−0.00906 (13)	0.2492 (4)	0.0414 (9)	
C11	0.3150 (2)	−0.04902 (13)	0.0609 (4)	0.0392 (9)	
C12	0.2159 (2)	−0.06559 (14)	−0.0565 (4)	0.0480 (10)	
C13	0.2145 (2)	−0.10416 (14)	−0.2344 (4)	0.0488 (10)	
C14	0.3122 (2)	−0.12898 (14)	−0.3082 (4)	0.0458 (10)	
C15	0.4128 (2)	−0.11114 (14)	−0.1943 (4)	0.0506 (11)	
C16	0.4124 (2)	−0.07274 (13)	−0.0153 (4)	0.0471 (10)	
C17	0.2077 (3)	−0.18754 (16)	−0.5982 (5)	0.0770 (16)	
C18	0.4122 (3)	−0.19039 (16)	−0.5680 (5)	0.0755 (15)	
H1	0.03700	0.11530	0.86510	0.0890*	
H1A	0.13160	0.06470	0.62090	0.0530*	
H2	0.03200	0.17960	1.16470	0.1050*	
H3	0.19290	0.22370	1.32310	0.0910*	
H4	0.35720	0.20510	1.18370	0.0900*	
H5	0.36550	0.13960	0.88690	0.0740*	
H10	0.39720	0.00050	0.30000	0.0500*	
H12	0.14890	−0.05000	−0.01300	0.0580*	
H13	0.14660	−0.11400	−0.30780	0.0590*	
H15	0.48010	−0.12540	−0.24010	0.0610*	
H16	0.48010	−0.06220	0.05780	0.0570*	
H17A	0.15590	−0.20220	−0.50190	0.1150*	
H17B	0.22150	−0.22340	−0.69260	0.1150*	
H17C	0.17720	−0.14990	−0.67680	0.1150*	
H18A	0.46280	−0.15340	−0.57210	0.1130*	
H18B	0.39490	−0.20720	−0.70750	0.1130*	
H18C	0.44600	−0.22520	−0.48100	0.1130*	

### Atomic displacement parameters (Å^2^)

**Table:**

	*U*^11^	*U*^22^	*U*^33^	*U*^12^	*U*^13^	*U*^23^
O1	0.0432 (13)	0.0847 (17)	0.0632 (13)	−0.0141 (12)	0.0010 (10)	−0.0260 (12)
N1	0.0437 (14)	0.0484 (15)	0.0409 (12)	−0.0052 (12)	0.0033 (11)	−0.0120 (11)
N2	0.0486 (17)	0.106 (2)	0.0607 (16)	−0.0128 (16)	0.0113 (13)	−0.0316 (15)
N3	0.0715 (19)	0.0641 (18)	0.0502 (14)	−0.0024 (15)	0.0063 (14)	−0.0211 (13)
C1	0.075 (2)	0.073 (2)	0.079 (2)	−0.031 (2)	0.035 (2)	−0.031 (2)
C2	0.108 (3)	0.075 (3)	0.088 (3)	−0.035 (2)	0.056 (2)	−0.036 (2)
C3	0.129 (4)	0.050 (2)	0.0486 (19)	−0.003 (2)	0.010 (2)	−0.0106 (16)
C4	0.087 (3)	0.068 (2)	0.066 (2)	0.019 (2)	−0.024 (2)	−0.0251 (19)
C5	0.061 (2)	0.059 (2)	0.0608 (19)	0.0158 (17)	−0.0159 (16)	−0.0210 (17)
C6	0.060 (2)	0.0341 (16)	0.0377 (14)	0.0019 (15)	0.0046 (15)	0.0007 (13)
C7	0.0429 (18)	0.0431 (18)	0.0412 (15)	−0.0066 (15)	0.0020 (14)	−0.0047 (13)
C8	0.0359 (16)	0.0451 (18)	0.0361 (13)	−0.0036 (13)	0.0013 (12)	0.0012 (12)
C9	0.0472 (19)	0.058 (2)	0.0396 (15)	−0.0055 (16)	0.0085 (14)	−0.0122 (14)
C10	0.0401 (16)	0.0427 (17)	0.0408 (14)	−0.0019 (14)	−0.0004 (13)	0.0003 (13)
C11	0.0377 (17)	0.0409 (17)	0.0387 (14)	−0.0017 (13)	0.0018 (13)	−0.0005 (13)
C12	0.0423 (18)	0.060 (2)	0.0425 (15)	0.0023 (15)	0.0080 (13)	−0.0058 (14)
C13	0.0477 (19)	0.058 (2)	0.0399 (15)	−0.0051 (15)	−0.0006 (13)	−0.0048 (14)
C14	0.058 (2)	0.0411 (18)	0.0389 (15)	−0.0003 (15)	0.0071 (15)	−0.0008 (13)
C15	0.051 (2)	0.049 (2)	0.0526 (17)	0.0034 (15)	0.0100 (15)	−0.0066 (15)
C16	0.0447 (18)	0.0462 (19)	0.0503 (16)	0.0012 (15)	0.0037 (14)	−0.0038 (14)
C17	0.093 (3)	0.079 (3)	0.057 (2)	−0.007 (2)	−0.0048 (19)	−0.0198 (18)
C18	0.098 (3)	0.073 (3)	0.0592 (19)	0.001 (2)	0.0285 (19)	−0.0163 (17)

### Geometric parameters (Å, °)

**Table:**

O1—C7	1.216 (3)	C12—C13	1.373 (4)
N1—C6	1.422 (3)	C13—C14	1.397 (4)
N1—C7	1.359 (3)	C14—C15	1.407 (4)
N2—C9	1.150 (4)	C15—C16	1.379 (4)
N3—C14	1.363 (4)	C1—H1	0.9300
N3—C17	1.445 (4)	C2—H2	0.9300
N3—C18	1.450 (4)	C3—H3	0.9300
N1—H1A	0.8600	C4—H4	0.9300
C1—C2	1.385 (5)	C5—H5	0.9300
C1—C6	1.375 (4)	C10—H10	0.9300
C2—C3	1.358 (6)	C12—H12	0.9300
C3—C4	1.349 (6)	C13—H13	0.9300
C4—C5	1.390 (4)	C15—H15	0.9300
C5—C6	1.370 (4)	C16—H16	0.9300
C7—C8	1.493 (4)	C17—H17A	0.9600
C8—C9	1.424 (4)	C17—H17B	0.9600
C8—C10	1.347 (4)	C17—H17C	0.9600
C10—C11	1.442 (4)	C18—H18A	0.9600
C11—C16	1.393 (3)	C18—H18B	0.9600
C11—C12	1.397 (4)	C18—H18C	0.9600
			
C6—N1—C7	128.4 (2)	C2—C1—H1	120.00
C14—N3—C17	121.5 (2)	C6—C1—H1	120.00
C14—N3—C18	122.2 (2)	C1—C2—H2	120.00
C17—N3—C18	116.3 (3)	C3—C2—H2	120.00
C6—N1—H1A	116.00	C2—C3—H3	120.00
C7—N1—H1A	116.00	C4—C3—H3	120.00
C2—C1—C6	120.6 (3)	C3—C4—H4	119.00
C1—C2—C3	119.9 (3)	C5—C4—H4	119.00
C2—C3—C4	119.7 (3)	C4—C5—H5	120.00
C3—C4—C5	121.3 (3)	C6—C5—H5	120.00
C4—C5—C6	119.4 (3)	C8—C10—H10	114.00
N1—C6—C5	124.7 (2)	C11—C10—H10	114.00
C1—C6—C5	119.0 (3)	C11—C12—H12	119.00
N1—C6—C1	116.3 (2)	C13—C12—H12	119.00
N1—C7—C8	114.8 (2)	C12—C13—H13	119.00
O1—C7—N1	124.0 (2)	C14—C13—H13	119.00
O1—C7—C8	121.2 (2)	C14—C15—H15	120.00
C9—C8—C10	122.4 (2)	C16—C15—H15	120.00
C7—C8—C10	119.9 (2)	C11—C16—H16	119.00
C7—C8—C9	117.7 (2)	C15—C16—H16	119.00
N2—C9—C8	177.1 (3)	N3—C17—H17A	109.00
C8—C10—C11	131.6 (2)	N3—C17—H17B	109.00
C12—C11—C16	116.1 (2)	N3—C17—H17C	109.00
C10—C11—C12	125.7 (2)	H17A—C17—H17B	109.00
C10—C11—C16	118.2 (2)	H17A—C17—H17C	109.00
C11—C12—C13	121.9 (2)	H17B—C17—H17C	110.00
C12—C13—C14	121.9 (2)	N3—C18—H18A	109.00
N3—C14—C15	121.3 (2)	N3—C18—H18B	109.00
N3—C14—C13	122.0 (2)	N3—C18—H18C	109.00
C13—C14—C15	116.8 (2)	H18A—C18—H18B	109.00
C14—C15—C16	120.5 (2)	H18A—C18—H18C	110.00
C11—C16—C15	122.9 (2)	H18B—C18—H18C	109.00
			
C6—N1—C7—O1	1.3 (4)	O1—C7—C8—C9	−175.1 (2)
C7—N1—C6—C1	179.4 (3)	O1—C7—C8—C10	4.5 (4)
C7—N1—C6—C5	−1.4 (4)	N1—C7—C8—C9	5.0 (3)
C6—N1—C7—C8	−178.8 (2)	C9—C8—C10—C11	2.3 (5)
C18—N3—C14—C15	−3.8 (4)	C7—C8—C10—C11	−177.2 (3)
C17—N3—C14—C13	−1.5 (4)	C8—C10—C11—C12	5.6 (5)
C18—N3—C14—C13	175.8 (3)	C8—C10—C11—C16	−175.5 (3)
C17—N3—C14—C15	179.0 (3)	C10—C11—C12—C13	−179.9 (3)
C2—C1—C6—N1	178.7 (3)	C16—C11—C12—C13	1.3 (4)
C2—C1—C6—C5	−0.6 (5)	C10—C11—C16—C15	−179.8 (2)
C6—C1—C2—C3	0.4 (5)	C12—C11—C16—C15	−0.8 (4)
C1—C2—C3—C4	0.3 (5)	C11—C12—C13—C14	−0.1 (4)
C2—C3—C4—C5	−0.8 (5)	C12—C13—C14—N3	178.9 (3)
C3—C4—C5—C6	0.7 (5)	C12—C13—C14—C15	−1.6 (4)
C4—C5—C6—N1	−179.1 (3)	N3—C14—C15—C16	−178.5 (3)
C4—C5—C6—C1	0.0 (4)	C13—C14—C15—C16	2.0 (4)
N1—C7—C8—C10	−175.5 (2)	C14—C15—C16—C11	−0.8 (4)

### Hydrogen-bond geometry (Å, °)

**Table:**

*D*—H···*A*	*D*—H	H···*A*	*D*···*A*	*D*—H···*A*
C5—H5···O1	0.93	2.30	2.892 (4)	121
C12—H12···N2	0.93	2.61	3.445 (4)	149
N1—H1A···N2^i^	0.86	2.50	3.245 (3)	146
C1—H1···N2^i^	0.93	2.58	3.338 (4)	139
C18—H18A···O1^ii^	0.96	2.49	3.439 (4)	169
C3—H3···Cg1^iii^	0.93	2.66	3.514 (3)	152

Symmetry codes: (i) −*x*, −*y*, −*z*+1; (ii) −*x*+1, −*y*, −*z*; (iii) *x*, −*y*+1/2, *z*+1/2.
